# Surface vimentin is critical for the cell entry of SARS-CoV

**DOI:** 10.1186/s12929-016-0234-7

**Published:** 2016-01-22

**Authors:** Yvonne Ting-Chun Yu, Ssu-Chia Chien, I-Yin Chen, Chia-Tsen Lai, Yeou-Guang Tsay, Shin C. Chang, Ming-Fu Chang

**Affiliations:** Institute of Biochemistry and Molecular Biology, College of Medicine, National Taiwan University, No. 1, Jen-Ai Road, First Section, Taipei, 100 Taiwan; Institute of Microbiology, College of Medicine, National Taiwan University, No. 1, Jen-Ai Road, First Section, Taipei, 100 Taiwan; Institute of Biochemistry and Molecular Biology, School of Life Sciences, National Yang-Ming University, Taipei, 112 Taiwan

**Keywords:** Severe acute respiratory syndrome, Angiotensin-converting enzyme 2, Virus entry, Spike protein, Vimentin

## Abstract

**Background:**

Severe acute respiratory syndrome coronavirus (SARS-CoV) caused a global panic due to its high morbidity and mortality during 2002 and 2003. Soon after the deadly disease outbreak, the angiotensin-converting enzyme 2 (ACE2) was identified as a functional cellular receptor in vitro and in vivo for SARS-CoV spike protein. However, ACE2 solely is not sufficient to allow host cells to become susceptible to SARS-CoV infection, and other host factors may be involved in SARS-CoV spike protein-ACE2 complex.

**Results:**

A host intracellular filamentous cytoskeletal protein vimentin was identified by immunoprecipitation and LC-MS/MS analysis following chemical cross-linking on Vero E6 cells that were pre-incubated with the SARS-CoV spike protein. Moreover, flow cytometry data demonstrated an increase of the cell surface vimentin level by 16.5 % after SARS-CoV permissive Vero E6 cells were treated with SARS-CoV virus-like particles (VLPs). A direct interaction between SARS-CoV spike protein and host surface vimentin was further confirmed by far-Western blotting. In addition, antibody neutralization assay and shRNA knockdown experiments indicated a vital role of vimentin in cell binding and uptake of SARS-CoV VLPs and the viral spike protein.

**Conclusions:**

A direct interaction between vimentin and SARS-CoV spike protein during viral entry was observed. Vimentin is a putative anti-viral drug target for preventing/reducing the susceptibility to SARS-CoV infection.

## Background

Severe acute respiratory syndrome (SARS), an atypical pneumonia, had emerged in late 2002 and spread to more than two dozen countries within Asia, South and North America, and Europe in the spring of 2003 [1]. During this period, the SARS epidemic had been considered as a significant threat to populations as its mortality rate was approximately 10 %, responsible for around 800 deaths out of approximately 8000 patients [2]. SARS-coronavirus (SARS-CoV), a novel coronavirus classified as a member of the *Coronaviridae* family was soon identified as the causative pathogen [3, 4]. The RNA genome of SARS-CoV consists of 14 potential major open reading frames that encode the viral non-structural proteins, accesory proteins, and structural proteins including spike (S), membrane (M), envelope (E), and nucleocapsid (N) proteins [5]. Angiotensin-converting enzyme 2 (ACE2), the type I integral transmembrane protein, was identified to be the functional receptor for SARS-CoV both in vitro [5, 6] and in vivo [7]. Studies on the expression of ACE2 protein and the tissue tropism and cellular distributions of SARS-CoV provided new insight into the mechanism of pathogenesis [8]. Nevertheless, certain ACE2-expressing endothelial cells and human intestinal cell lines failed to be infected by SARS-CoV [9, 10]. In contrast, cells without a detectable expression level of ACE2 such as hepatocytes could be infected by SARS-CoV [8]. In addition, the presence of ACE2 alone is not sufficient for maintaining viral infection [8]. Altogether, these observations indicate that different virus receptors or co-receptors may be utilized in the infection of SARS-CoV in various tissues. Indeed, DC-SIGN, a c-type lectin receptor expressed on dendritic cells, and a DC-SIGN-related molecule, L-SIGN (also named DC-SIGNR and CD209L) have been indicated to interact with the SARS-CoV spike protein and to facilitate the virus dissemination [11, 12].

Vimentin is the major component of the type III intermediate filament protein which aims mainly to maintain the architecture of cytoplasm, it can also be secreted under certain conditions [13]. Vimentin participates in cell adhesion, migration, and cellular signaling [14, 15]. In addition, vimentin has been reported to play roles in viral multiplication. Rearrangement of cytosolic vimentin and formation of vimentin cages around the viral factories were observed during the infection of vaccinia virus and African swine fever virus [16, 17]. Studies on mammalian porcine reproductive and respiratory syndrome virus, Japanese encephalitis virus, and cowpea mosaic virus also provided evidence that binding of virus to surface vimentin can facilitate internalization and infection [18–21]. Blocking the expression and binding of surface vimentin inhibited viral entry.

In this study, intermediate filament vimentin was identified as a cellular factor abundantly present in the SARS-CoV spike protein-ACE2 complexes. Incubating Vero E6 cells with SARS-CoV virus-like particles (VLPs) enhanced the expression level of the surface form of vimentin. Co-localization of vimentin and the SARS-CoV spike protein was observed in a short time period soon after incubation. Further studies indicate that vimentin directly binds to the SARS-CoV spike protein and is involved in the entry of SARS-CoV. These results suggest that vimentin serves as a putative co-receptor for coordinately interacting with ACE2 during SARS-CoV infection. The study provides a new target for drug development against SARS-CoV infection.

## Methods

### Cell lines

*Sf9* (*Spodoptera frugiperda*) insect cells were maintained at 27 °C while being supplied with Sf-900IISFM medium (Gibco). Vero E6 cells (African green monkey kidney epithelial cells) were maintained at 37 °C with 5 % CO_2_ in Dulbecco’s modified Eagle’s medium (Gibco), supplemented with heat-inactivated fetal bovine serum (8 %), penicillin (100 units/ml), and streptomycin (100 μg/ml).

### Production and purification of SARS-CoV VLPs and the SARS-CoV spike protein

Production and purification of SARS-CoV VLPs and SARS-CoV spike protein were carried out as described previously [22]. In brief, *Sf*9 cells were coinfected with three recombinant baculoviruses expressing V5-tagged SARS-CoV E, M, and S proteins individually. Sf-900IISFM conditional medium containing properly assembled VLPs was harvested at 4 days post-infection. SARS-CoV VLPs were then purified by sucrose gradient centrifugation at 26,700 rpm for 3.5 h at 4 °C using an SW41 rotor in a Beckman L9-M Ultracentrifuge. SARS-CoV VLPs were analyzed by immunogold electron microscopy (data not shown). In addition, the number of SARS-CoV VLPs was calculated by atomic force microscopy as described previously [22]. To obtain the SARS-CoV spike protein, a culture medium of *Sf9* cells previously infected with the recombinant baculoviruses expressing the C-terminal V5- and His-tagged full-length SARS-CoV spike protein were collected at 4 days post-infection and subjected to the purification of the spike protein by using a Ni^2+^ Sepharose purification system.

### Extracellular chemical cross-linking

Extracellular chemical cross-linking of Vero E6 cells pre-incubated with SARS-CoV VLPs at VLP-to-cell ratio 1000:1 was performed at 4 °C for 2 h with 5 mM membrane-impermeant primary amine-reactive cross-linker, bis(sulfo-succinimidyl) suberate (BS^3^, Pierce) or the thiol-cleavable reagent 3,3’-dithiobis(sulfo-succinimidylpropionate) (DTSSP, Pierce) in the reaction buffer (20 mM Na_3_PO_4_ and 0.15 M NaCl in PBS, pH 8.0). The reaction mixtures were then quenched with 20 mM Tris buffer (pH 7.5) for 15 min at room temperature and subjected to cell lysis for further identification of ACE2-associated proteins.

### Immunoprecipitation, silver staining, and mass spectrometry

Following extracellular chemical cross-linking, the SARS-CoV VLP-pre-incubated Vero E6 cells were lysed with Empigen BB lysis buffer (50 mM Tris-HCl, pH 7.4, 0.05 % sodium deoxycholate, 150 mM NaCl, 1 mM EDTA, and 0.3 % Empigen BB) supplemented with protease inhibitor cocktail (Roche) and phenylmethylsulfonyl fluoride. The protein lysates were subjected to immunoprecipitation followed by sodium dodecyl sulfate-polyacrylamide gel electrophoresis (SDS-PAGE), silver staining, and LC-MS/MS analysis as described previously [23, 24].

### Flow cytometry

Flow cytometry analysis was performed for quantitative determination of cell surface vimentin and VLP-positive or spike-positive cells. For the detection of surface vimentin, Vero E6 cells were pre-incubated with SARS-CoV VLPs at VLP-to-cell ratio 100:1 for 10 min. Cells were subsequently fixed with 4 % formaldehyde in FACS buffer (5 mM EDTA in PBS, pH 7.4) for 20 min followed by incubation with mouse anti-vimentin antibodies (V5255, Sigma) and the goat anti-mouse AlexaFluor 488-conjugated antibodies. For quantitative determination of VLP-positive cells and spike-positive cells, Vero E6 cells pre-incubated with SARS-CoV VLPs or purified spike protein were fixed in FACS buffer with 4 % formaldehyde, permeabilized in FACS buffer with 0.1 % Triton X-100, and subjected to immunofluorescence labeling with goat anti-mouse AlexaFluor 594-conjugated antibodies following incubating with anti-V5 epitope antibodies (Invitrogen) and with goat anti-rabbit AlexaFluor 488-conjugated antibodies following incubating with rabbit anti-6-His antibody (Bethyl Laboratories Inc.), respectively. The immunofluorescence stained cells were subjected to LSR-II Digital Flow Cytometer (BD Biosciences) and the data was analyzed using FlowJo software (Tree Star Inc.). In antibody neutralization experiments, rabbit monoclonal anti-vimentin antibody (RP4002, Immuno Bioscience Corp.), mouse monoclonal anti-vimentin antibodies (V5255, Sigma) and goat polyclonal anti-ACE2 antibodies (AF933, R&D Systems) were applied prior to the treatment of SARS-CoV VLPs or spike protein and subsequent flow cytometry. In vimentin knockdown experiments, Vero E6 cells were infected by lentiviruses carrying shRNAs specific to vimentin prior to the treatment of spike protein and subsequent flow cytometry.

### Confocal microscopy and immunofluorescence staining assay

Cells grown on 18 mm round glass coverslips were fixed using 4 % formaldehyde in PBS for 12 min. Then the cells were rinsed with PBS buffer, subsequently an appropriate amount of 1:1 of acetone/methanol solution was added and incubated for 2 min to permeabilize the cell membranes. PBS buffer was immediately applied followed by quick rinse using PBS buffer. In the study for monitoring the proteins on cellular membranes, the permeabilization step was skipped. The cells were treated with 4 % BSA in PBS buffer to block for 1 h, then 1:10,000 of primary antibodies in PBS buffer containing 1 % BSA at 4 °C overnight. Next day, the cells were rinsed with PBS, and subsequently incubated with secondary antibodies at 1:5000 in PBS buffer containing 1 % BSA for 1 h at room temperature. To stain the nuclei, 1:500 of Hoechst in PBS buffer containing 1 % BSA was added at the same time as the addition of secondary antibodies. The coverslips were then treated with mounting fluids and sealed for observation. For certain groups of experiments aiming to examine surface vimentin upon SARS-CoV VLPs binding, Vero E6 cells were pre-incubated with SARS-CoV VLPs at VLP-to-cell ratio 100:1. The images were acquired on a *Leica TCS SP5 Confocal Microscopy*. For immunofluorescence staining assay, Vero E6 cells pre-incubated with SARS-CoV VLPs were fixed and subjected to analysis as described previously [23] using mouse anti-vimentin antibodies (Sigma) and rabbit anti-SARS-CoV spike protein antibodies (IMGENEX) as the primary antibodies, and AlexaFluor 488-conjugated goat anti-mouse IgG antibody and AlexaFluor 594-conjugated goat anti-rabbit IgG antibody, respectively, as the secondary antibodies.

### Far-Western blot analysis and Western blot analysis

For far-Western blot analysis, cell lysates resolved by SDS-PAGE were transferred onto a PVDF membrane. Following a blocking with 3 % BSA in PBS buffer, the membrane was then incubated with purified SARS-CoV spike protein (10 μg/ml) at 4 °C overnight in PBS buffer containing 1 % BSA. Detection of the binding of the purified spike protein, along with its interacting cellular proteins resolved on the membrane were detected by Western blot analysis as described previously [25].

### Lentivirus preparation and infection

The lentivirus vector pLKO.1 carrying the small hairpin RNA (shRNA) targeting vimentin (shVim-A: 5‘-GCATCACGATGACCTTGAATA-3’, TRCN0000089830; shVim-B: 5‘-GCTAACTACCAAGACACTATT-3’, TRCN0000029119; shVim-C: 5‘-GCGCAAGATAGATTTGGAATA-3’, TRCN0000089828) and targeting luciferase (shLuc: 5‘- CAAATCACAGAATCGTCGTAT-3’, TRCN0000072246) from the National RNAi Core Facility, Academia Sinica, Taiwan were individually cotransfected with pMD.G and pCMVΔR8.91 to HEK293T cells by using T-Pro NTR II transfection reagent (T-Pro Biotechnology). The culture supernatants containing lentiviruses carrying individual shRNAs were then harvested and used to knockdown vimentin expression in Vero E6 cells following infection. In addition, polybrene (Sigma) was added at 8 μg/ml to increase efficiency of infection. The lentivirus carrying shRNA against luciferase served as a negative control in vimentin knockdown experiments.

## Results

### Vimentin is present in SARS-CoV spike protein-ACE2 complex

**Fig. 1:**
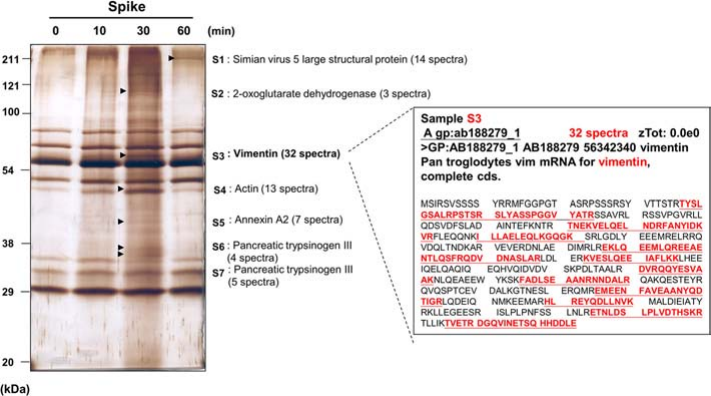
Vimentin as a cross-linked protein in association with the SARS-CoV spike protein-ACE2 complex. Vero E6 cells at 8 × 10^6^ were pre-incubated with SARS-CoV spike protein (1 μg/10^6^ cells) at 4 °C for various time periods as indicated followed by a treatment with the thiol-cleavable cross-linker DTSSP. Cell lysates were harvested and subjected to immunoprecipitation with anti-ACE2 antibodies. Vero E6 cells being treated by SARS-CoV VLPs for 0 min was used as the negative control. The immunoprecipitates were incubated with protein sample buffer with the addition of 5 % β-mercaptoethenol and subjected to SDS-PAGE. Proteins separated on the polyacrylamide gel were visualized by silver staining. The protein bands marked S1 to S7 were collected for LC-MS/MS analysis and results were shown. The number of matched spectra of the protein fragments compared to the published data was stated in parentheses. The detailed analysis of S3 was shown in the inset, where sequence comparison with data banks indicated the S3 protein to be vimentin. Underlines show the peptide sequences of 32 spectra

To examine the putative SARS-CoV spike protein-interacting host factors, SARS-CoV-permissive Vero E6 cells were incubated with SARS-CoV VLPs in the presence of membrane-impermeant BS^3^ that triggered a chemical cross-link of the N-terminal domain of SARS-CoV spike protein to its functional receptor ACE2 as well as other components appear in the spike-receptor complexes. While Vero E6 cell lysates were immunoprecipitated by anti-ACE2 antibodies, those pre-treated with purified SARS-CoV spike proteins were shown to form various complexes with molecular sizes larger than 200 kDa (data not shown). This is an indication of numerous host factors associated with viral spike-ACE2 complexes upon binding. To further dissect which proteins were involved in the complexes, the reversible cross-linker DTSSP was used to link interacting partners within 12 Å. Subsequently, DTSSP-linked proteins were cleaved on the central disulfide bonds of the cross-linkers by adding the reducing agent β-mercaptoethanol prior to SDS-PAGE followed by silver staining. As a result, protein bands seen on the gel referred to the molecular weight of each component in the spike-ACE2 complex. By comparing the Vero E6 cell lysates pre-incubated with SARS-CoV VLPs in a time course from 10, 30, to 60 min with those treated VLPs for 0 min as a reference, seven major protein bands, named S1 to S7 (indicated by black arrowheads), showed differential abundance (Fig. 1). The bands were then cut off the gel and sent for LC-MS/MS analysis. The resulting spectra of protein S3 demonstrated that 32 fragments were matched to a fraction of vimentin protein sequence, suggesting that a cytoskeletal protein vimentin is one of the putative ACE2-interacting proteins (inset in Fig. 1).

**Fig. 2:**
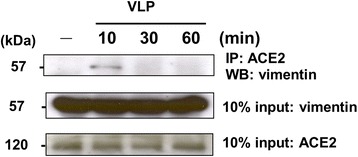
Vimentin involved in the SARS-CoV spike-ACE2 complex on plasma membrane at different time points. Vero E6 cells were incubated with SARS-CoV VLPs for 10, 30 and 60 min at 37 °C. Cell lysates were then prepared and subjected to immunoprecipitation (IP) with anti-ACE2 antibodies, followed by Western blot (WB) analysis with anti-vimentin antibodies (Sigma). Cell lysates without the process of immunoprecipitation (10 % input) were analyzed in parallel with anti-vimentin and anti-ACE2 antibodies as the protein loading controls

To further confirm the interaction between vimentin and the ACE2 protein, cell lysates prepared from SARS-CoV VLP-treated Vero E6 cells were subjected to co-immunoprecipitation with anti-ACE2 antibodies and detected with anti-vimentin antibodies. The negative control for this experiment was Vero E6 cells without the treatment of SARS-CoV VLPs. As shown in Fig. 2, vimentin formed complex with ACE2 in Vero E6 cells pretreated with the SARS-CoV VLPs for 10 min. Nevertheless, the interaction between vimentin and spike-ACE2 complex on the membrane is transient as co-immunoprecipitation of vimentin was no longer detected after 10 min. These results suggest an involvement of the cell surface vimentin in the virus entry via an interaction, for a relatively short time period soon after exposing to the SARS-CoV VLPs, with the spike-ACE2 complexes. Speculation regarding the conflicts about whether vimentin interacted with ACE2 at 30 min (Fig. 1) or 10 min (Fig. 2) will be given in the Discussion section.

### The expression of cell surface form of vimentin is induced by SARS-CoV VLPs

**Fig. 3:**
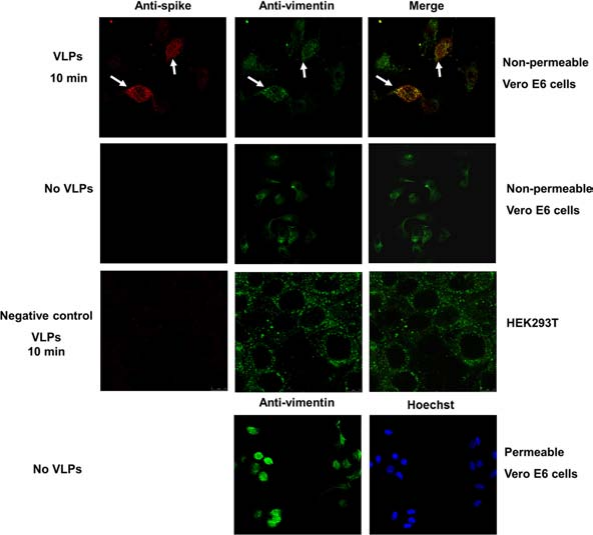
Co-localization of the spike protein with vimentin in Vero E6 cells pre-treated with SARS-CoV VLPs. Vero E6 cells pre-treated with SARS-CoV VLPs for 10 min were fixed for immunofluorescence assay with anti-spike antibodies followed by AlexaFluor 594-conjugated goat anti-rabbit IgG and anti-vimentin antibodies followed by AlexaFluor 488-conjugated goat anti-mouse IgG antibodies as shown in red and green colors, respectively. Vero E6 cells without the pretreatment with SARS-CoV VLPs and ACE2-negative HEK293T cells treated with SARS-CoV VLPs for 10 min were used as controls. Hoechst staining was performed in parallel to localize cell nuclei in the field and the distribution of vimentin in cytosol was examined using permeable Vero E6 cells

To further examine the biological role of vimentin in association with the spike-ACE2 complexes, the expression of vimentin and its cellular distribution were examined by immunofluorescence assay following a pretreatment of Vero E6 cells with the SARS-CoV VLPs for 10 min. As shown in Fig. 3, vimentin colocalized with the SARS-CoV spike protein on the cell surface (indicated by white arrows) under a non-permeable fixation condition that allowed only the detection of the protein level on the extracellular side. For the negative control, Vero E6 cells without pretreatment with the SARS-CoV VLPs were shown no detectable signals referring to spike proteins on the membrane. Although some background noise appeared when the anti-vimentin antibody was applied to detect surface vimentin, no co-localization of spike and vimentin can be observed. In addition, HEK293T cell line was used to ensure that, when there was a lack of ACE2, the cells cannot accommodate the binding of spike proteins on the membranes; moreover, vimentin showed no correlation with spike-ACE2 complex in terms of the distributions (Fig. 3). When Vero E6 cells without pretreatment of SARS-CoV VLPs were permeabilized by 1:1 acetone/methanol, vimentin protein was detected in the cytosols.

**Fig. 4:**
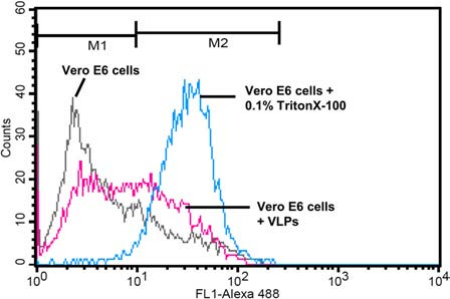
An increased level of cell surface vimentin induced by SARS-CoV VLPs. Vero E6 cells suspended in FACS buffer were incubated with SARS-CoV VLPs for 10 min at 4 °C. The cells were then fixed and analyzed with anti-vimentin antibodies and AlexaFluor 488-conjugated secondary antibodies. The fluorescence was quantified by using a LSR-II Digital Flow Cytometer and the data were analyzed by using FlowJo software. Vero E6 cells without the pre-treatment of SARS-CoV VLPs were used as a negative control (M1 region) and Vero E6 cells permeabilized with 0.1 % TritonX-100 for 5 min were used as a positive control (M2 region)

To quantify the amount of surface vimentin induced by SARS-CoV VLPs treatment, flow cytometry was utilized. Using Vero E6 cells permeabilized with 0.1 % Triton X-100 for the detection of intracellular vimentin as a positive control (light blue peak), an increase of 16.5 % on the cell surface vimentin upon a 10-min application of SARS-CoV VLPs (magenta curve) was detected in comparison with those without the VLP-pretreatment (grey peak) (Fig. 4).

**Fig. 5:**
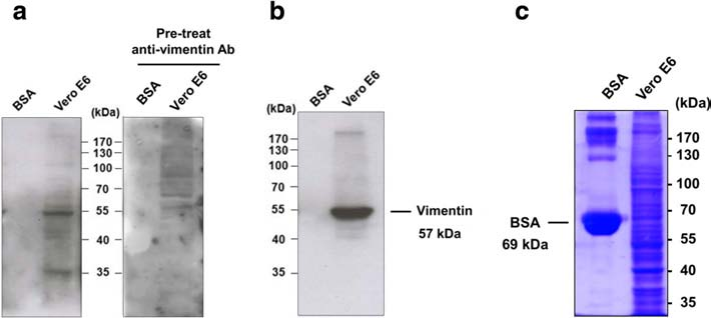
Direct interaction between vimentin and SARS-CoV spike protein analyzed by far-Western blot analysis. **a** Far-Western blot analysis. Protein lysates prepared from Vero E6 cells were blotted on PVDF membrane and subjected to far-Western blot analysis with anti-V5 epitope antibodies (*left panel*) following a pre-incubation of the membrane with the purified V5-tagged recombinant SARS-CoV spike protein. The right panel showed the Vero E6 cells pre-incubated with anti-vimentin antibodies (Sigma) prior to the treatment of SARS-CoV spike protein. **b** Western blot analysis. The same set of protein lysates blotted on PVDF membrane was subjected to Western blot analysis with anti-vimentin antibodies to serve as a positive control. **c** Coomassie blue staining. Coomassie blue staining showed the total proteins on the gel. BSA loaded in parallel was used as a negative control

Knowing that vimentin is present in the viral spike protein-ACE2 complex and potentially important for the cell entry of SARS-CoV VLPs, it is vital to understand if the interaction between surface vimentin and spike or ACE2 is direct or indirect. By conducting far-Western blot analysis, a direct interaction between the viral spike protein and vimentin was then concluded from Fig. 5. The purified V5-tagged spike protein colocalized with the 57 kDa vimentin blotted on the membrane, indicating a direct binding of the spike protein to vimentin (Fig. 5a left panel). Besides, while the membrane was pre-treated with anti-vimentin antibodies prior to spike protein incubation, vimentin cannot be detected via far-Western blotting (Fig. 5a right panel). Western blot analysis with antibodies to vimentin was performed in parallel as a positive control (Fig. 5b). In addition, a Coomassie Blue staining was shown in Fig. 5c as an internal control with the aim of ensuring there are no any relatively abundant cellular proteins of 57 kDa which might interfere with far-Western blot analysis. Taken together, these data suggest an involvement of the surface vimentin in the virus entry via interacting with the spike-ACE2 complexes.

### Vimentin is involved in the entry process of SARS-CoV VLPs

**Fig. 6:**
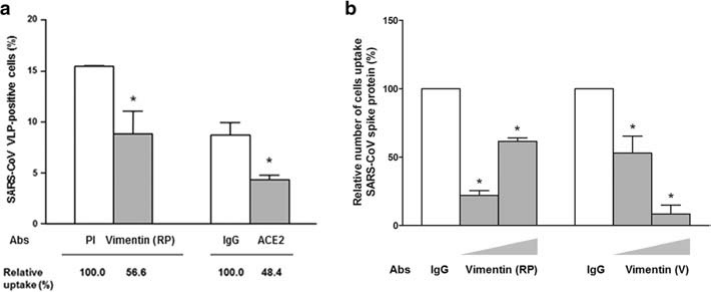
Anti-vimentin antibodies specifically diminished the uptake of SARS-CoV VLPs and spike protein by Vero E6 cells. **a** The effect of anti-vimentin antibodies and anti-ACE2 antibodies on the uptake of SARS-CoV VLPs. Vero E6 cells were pretreated with rabbit anti-vimentin antibodies RP (RP4002, Immuno Bioscience Corp.) or goat anti-ACE2 antibodies for 30 min prior to the 2-h incubation with SARS-CoV VLPs. The cells were then subjected to flow cytometry analysis for quantitation of the SARS-CoV VLP-positive cells as described in Methods. Rabbit pre-immune serum (PI), and goat IgG as indicated were used as antibody isotype controls in the neutralization experiments. The number of cells pretreated with isotype antibodies was normalized to 100 %, subsequently the relative uptake of SARS-CoV VLPs after antibody neutralization was calculated and shown underneath the bar chart. **b** The effect of anti-vimentin antibodies on the uptake of spike protein. Vero E6 cells were pretreated with increasing doses of rabbit anti-vimentin antibodies RP (RP4002, Immuno Bioscience Corp.) or mouse anti-vimentin antibodies V (V5255, Sigma) as indicated for 30 min prior to the 2-h incubation with SARS-CoV spike protein. SARS-CoV spike-positive cells were then quantitated by flow cytometry analysis. The number of cells uptake SARS-CoV spike protein after antibody neutralization was calculated and normalized against the number of cells pretreated with IgG control in each set of experiment. * indicates the *P* value < 0.05

**Fig. 7:**
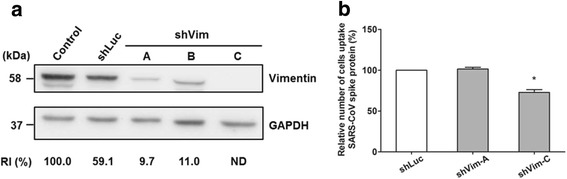
Knockdown of vimentin expression to an undetectable level specifically reduced the uptake of SARS-CoV spike protein by Vero E6 cells. **a** Lentivirus-mediated knockdown of vimentin. Vero E6 cells infected by lentiviruses carrying shRNAs specific to vimentin (shVim-A, B, and C) were harvested at 4 days postinfection for Western blot analysis with antibodies against vimentin (Sigma). Cells without infection (Control) and cells infected by lentiviruses carrying shRNA against luciferase (shLuc) served as controls. GAPDH was used as an internal control. RI: relative intensity; ND: not detectable. **b** The effect of vimentin knockdown on the uptake of SARS-CoV spike protein. Vero E6 cells were infected by lentiviruses carrying shRNAs as indicated for 4 days prior to the 2-h incubation with SARS-CoV spike proteins. The cells were then subjected to flow cytometry analysis for quantitation of the SARS-CoV spike-positive cells. The number of cells uptake SARS-CoV spike proteins after vimentin knockdown was calculated and normalized to the number of cells pretreated with lentiviruses carrying shRNA against luciferase (shLuc). * indicates the *P* value < 0.05

The specificity of the surface vimentin involving in the entry of SARS-CoV VLPs was further elucidated by antibody neutralization experiments. Vero E6 cells were pre-incubated with anti-vimentin antibodies (RP), anti-ACE2 antibodies or control antibodies prior to the treatment of SARS-CoV VLPs. Intracellular VLPs were monitored by flow cytometry using anti-V5 antibodies that detected membrane-permeabilized SARS-CoV VLP-positive Vero E6 cells. Results demonstrated a 43.4 % reduction (15.41 ± 0.083 vs. 8.73 ± 2.127 %) on the number of cells that uptook SARS-CoV VLPs when the cells were pre-treated with anti-vimentin antibodies, while cells pre-treated with anti-ACE2 antibodies showed a 51.6 % decline (8.86 ± 1.135 vs. 4.25 ± 0.541 %) (Fig. 6a). The results suggested that anti-vimentin antibodies successfully blocked SARS-CoV VLPs entering into Vero E6 cells and its neutralization efficiency was comparable to that of the anti-ACE2 antibody. An inhibitory effect of anti-vimentin antibodies was also observed on the uptake of SARS-CoV spike protein. When Vero E6 cells were pre-treated with anti-vimentin antibodies RP at two different doses, a 78.0 and 38.6 % reduction on the number of cells that uptook SARS-CoV spike protein were observed, respectively; while cells pre-treated with different doses of anti-vimentin antibodies V showed a 47.0 and 91.6 % decline, respectively (Fig. 6b). The results further confirmed that vimentin is critical for the entry of SARS-CoV. The role of vimentin in the cell entry of SARS-CoV was also evident by knockdown experiments. Vero E6 cells infected by lentiviruses carrying shRNA specific to vimentin (shVim-A, shVim-B and shVim-C) displayed remarkable reductions on the protein expression of vimentin (Fig. 7a). When cells were pre-infected by lentiviruses carrying shVim-C, which reduced expression of vimentin protein to an undetectable level, a 27.2 % reduction on the uptake of SARS-CoV spike protein was observed (Fig. 7b). Altogether, surface vimentin interacts directly with the viral spike protein in a spike-ACE2 complex on Vero E6 cell membrane and is involved in the entry process of SARS-CoV. In addition, since shVim-A which knocked-down vimentin expression to about 10 % had little effect on the uptake of spike protein, a low level of vimentin protein may already sufficient to facilitate the entry of SARS-CoV.

## Discussion

Previous studies demonstrated that cell fusion occurred at 37 °C at roughly 1 h after coculturing the viral spike-expressing 293 T cells and cellular receptor ACE2-expressing Vero E6 cells [26]. By using membrane-insoluble cross-linkers BS^3^ and DTSSP to capture interacting proteins in close proximity (within 12 Å) in the current study, vimentin was detected at 30 min after the treatment of Vero E6 cells with purified viral spike protein (Fig. 1). The follow-up experiment demonstrated the interaction of SARS-CoV VLPs with the receptor ACE2 within 10 min of incubation (Fig. 2). Since the structure of SARS-CoV VLPs is similar to that of SARS-CoV with a crown-shaped appearance and about 100 nm in diameter [22], it is proposed that binding topology of the conformation-mimicking spike protein of VLPs to its ACE2 receptor is similar to the topology of SARS-CoV spike protein and requires a shorter period of time than the partially purified spike protein. Apart from the aforementioned reason, the different experimental conditions might also be playing a significant role on when vimentin proteins appear on cellular membranes since the cells treated with the purified SARS-CoV spike protein have a reacting temperature of 4 °C (Fig. 1), whereas those treated with SARS-CoV VLPs were conducted at 37 °C (Fig. 2).

Previous pathological studies indicated that alveolar epithelial cells are the essential cell type involved in SARS infection, and subsequent respiratory failure. Since the alveolar epithelium cells can further differentiate into type I and type II cells, both cell types are theoretical targets for SARS-CoV infections. Nevertheless, ACE2 had been proven to be largely expressed in the type II cells of the human lung [27–29] which was thus believed to be the primary target for SARS-CoV [30, 31]. This proposal was supported by another in vitro study in which human alveolar type II cells were demonstrated to be susceptible to the SARS nucleocapsid (SARS-N) protein, even though immunofluorescence staining failed to detect vimentin expression in these type II cells [32]. In the above mentioned study, infection of SARS-CoV was also detected in the primary culture of type I-like cells, where replication could not be successfully carried out. In addition, the studies on pulmonary stem/progenitor cells demonstrated their capability of differentiating into alveolar type II- and type I-like pneumocytes sequentially, both cell types were susceptible to SARS-CoV infection [33]. On the other hand, clinical and in vivo studies using macaque monkey models indicated that the target cells of SARS-CoV are primarily type I pneumocytes [34]. These studies stated above reveal that the susceptibility of either type I or type II alveolar cells to SARS-CoV infection is still controversial among different species. On top of this, the mechanism of stem/progenitor cell differentiation to the two types of alveolar cells is not yet clear. Nevertheless, our studies support the role of vimentin in the cell entry of SARS-CoV.

Our data also provided the evidence that SARS-CoV VLPs enhanced the expression of vimentin on the cell membrane. This is the first observation towards the impact of vimentin on SARS-CoV cell entry, although the potential extracellular and surface-associated functions of vimentin have been reported previously in the Mycobacterium tuberculosis-infected monocytes, where vimentin acts as a ligand for the NKp46 receptor on natural killer cells [35]. Besides, one previous study demonstrated that the tail region of surface vimentin was the endothelial target for Cowpea mosaic virus (CPMV) during infection [21], supporting the hypothesis that vimentin is functionally important in the viral pathogenesis. Furthermore, the association of vimentin with the outer capsid protein VP2 was also considered essential for bluetongue virus egress [36]. One more example in African swine fever virus provided more details in the mechanism of vimentin during viral pathogenesis, suggesting that phosphorylation and rearrangement of vimentin occurred after virus infection. A vimentin cage was then subsequently formed around the virus assembly site during the later stages of infection [16]. In this research, vimentin is one of the interacting partners of the ACE2 receptor and has direct contact with SARS-CoV spike protein. Yet, which domain of vimentin plays the role of interacting with viral spike protein remains unclear. Moreover, whether vimentin binds to ACE2 directly or they only arrange in close proximity will need to be further confirmed. As mentioned, the tail region of vimentin with a unique conformation was shown to be important for CPMV entry [21] and a potential association with the membrane proteins. Hence, it is hypothesized that a putative SARS-CoV spike protein-binding domain may reside in the tail region of vimentin.

Despite the fact that vimentin was known as intracellular filamentous cytoskeletal protein [37, 38], it has recently been indicated to be secreted from blood endothelial cells and activated macrophages through the Golgi apparatus and present on the plasma membrane [13]. Previous studies also suggested that vimentin may be localized on the cell membrane due to its structure with highly positively charged amino-terminal domain and a Tyr-X-X-Y motif (where X is any amino acid, and Y is a bulky hydrophobic amino acid) followed by a di-acidic motif (Asp-X-Glu)-containing carboxy terminus [39]. Furthermore, phosphorylation of vimentin by several protein kinases has been identified [40, 41]. The secreted vimentin has a higher level of phosphorylation and a lower apparent molar mass than the intracellular form [13]. The evidence stated above implies that secreted and intracellular vimentin may have differential biological functions.

It has previously been demonstrated in our laboratory that the SARS-CoV spike protein stimulates the activation of Ras-ERK-AP-1 signaling pathway and the expression of fibrosis-associated CCL2 through the phosphorylation of ACE2 [22]. Also, vimentin was identified as a β-adrenergic receptor (βAR)-interacting protein, which involved in the Src to ERK signaling pathway [42]. It is proposed that the rearrangement of vimentin from intracellular compartments to the cell surface might be resulted from the phosphorylation of vimentin through the activation of Ras-ERK signaling pathway induced by the interaction between SARS-CoV spike protein and ACE2. The cell surface vimentin further participates in the viral entry by associating with the spike-ACE2 complexes. However, whether vimentin is involved in the activation of ACE2 downstream signaling pathways needs to be further investigated.

## Conclusions

**Fig. 8:**
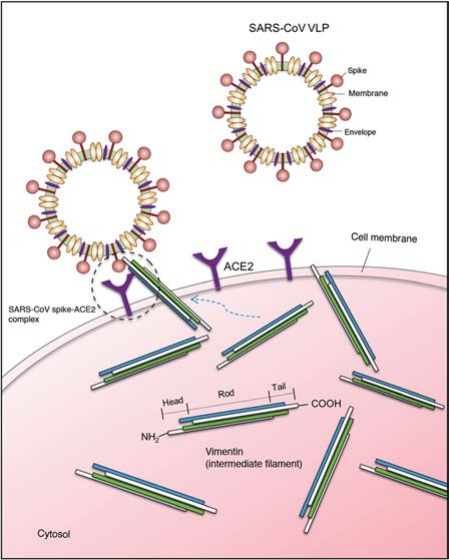
Simplified diagram of the involvement of vimentin within SARS-CoV spike-ACE2 complex during cell entry. The dashed circle indicates the spike-ACE2 complex on the cellular membrane, and the blue dotted arrow demonstrates the possible pathway when vimentin translocates from cytosol to membrane

In this study, we have demonstrated that cytoplasmic vimentin was translocated to the cell surface and interacted with the spike-ACE2 complex, revealing a putative entry pathway for SARS-CoV (Fig. 8). This study is the first report indicating that vimentin interacts directly with SARS-CoV spike protein during the spike-ACE2 binding process and serves as a putative co-receptor involved in the entry of SARS-CoV. Ultimately, the results may help in the development of new drugs for preventing the infection of SARS-CoV or reducing the susceptibility to SARS-CoV infection.

## Abbreviations

SARS-CoV: severe acute respiratory syndrome coronavirus
ACE2: angiotensin-converting enzyme 2
VLP: virus-like particle
